# Supplementary material for: Integrative models of histopathological images and multi-omics data predict prognosis in endometrial carcinoma
Source: PeerJ. 2023 Aug 11;11:e15674. doi: 10.7717/peerj.15674 (PMC10424667; doi:10.7717/peerj.15674)
Supplement: Supplemental Information 1 — 735 hematoxylin and eosin (H&E)-stained histopathological images of 465 patients from The Cancer Imaging Archive (TCIA). [file peerj-11-15674-s001.docx]

| **Characteristics** | TCGA-LUAD | | | P |
| --- | --- | --- | --- | --- |
|  | Training set (n=215) | Test set  (n= 214) | |  |
| Age: mean ± SD | 63.9±11.0 | | 64.2±10.7 | 0.797 |
| **Tumor stage (%)** |  | |  |  |
| I | 130 (60.5%) | | 138(64.5%) |  |
| II | 21(9.8%) | | 18(8.4%) |  |
| III | 49(22.8%) | | 49(22.9%) |  |
| IV | 15(7.0%) | | 9(4.2%) | 0.579 |
| **Histological grade (%)** |  | |  |  |
| G1 | 37(17.2%) | | 38(17.8%) |  |
| G2 | 49(22.8%) | | 47(22.0%) |  |
| G3 | 126(58.6%) | | 125(58.4%) |  |
| High Grade | 3(1.4%) | | 4(1.9%) | 0.989 |
| **Survival status (%)** |  | |  |  |
| Alive | 178(83.8%) | | 183(85.5%) | 0.440 |
| Deceased | 37(12.2%) | | 31(14.5%) |  |
| **Histological type (%)** |  | |  |  |
| Serous endometrial adenocarcinoma | 37(17.2%) | | 43（20.1%） |  |
| Endometrioid endometrial adenocarcinoma | 169（78.6%） | | 163（76.2%） |  |
| Mixed serous and endometrioid | 9（4.2%） | | 8（3.7%） | 0.735 |

Table S1: Demographic and clinical characteristics of patients.
