# Supplementary material for: Integrative models of histopathological images and multi-omics data predict prognosis in endometrial carcinoma
Source: PeerJ. 2023 Aug 11;11:e15674. doi: 10.7717/peerj.15674 (PMC10424667; doi:10.7717/peerj.15674)
Supplement: Supplemental Information 2 — The RF models were impressive in predicting common gene aberrations in EC, as they achieved relatively high AUC. [file peerj-11-15674-s002.docx]

GBDT CN_high CN_low MSI PLOE CTNNB1 PIK3CA PIK3R1 PTEN

RF 0.89599135 0.89599135 0.849369279 0.812467998 0.868792017 0.822815636 0.8255104 0.787225357

ADABAG 0.819556697 0.819556697 0.75492163 0.744623656 0.757563025 0.703161381 0.7328 0.693826751

DT 0.718326801 0.718326801 0.644075235 0.602150538 0.657773109 0.605891208 0.641066667 0.60066379

GBDT 0.836183268 0.836183268 0.810788715 0.851862519 0.768787515 0.704397832 0.7275296 0.67355349

LR 0.727682119 0.727682119 0.723435737 0.671317631 0.642839136 0.585809916 0.6285696 0.567911273

nNB 0.676888769 0.676888769 0.622633229 0.711981567 0.592279412 0.571698113 0.550533333 0.541232437

SVM 0.639626977 0.639626977 0.66539185 0.611239119 0.557930672 0.552358491 0.597866667 0.534760482

KNN 0.643249088 0.643249088 0.510542947 0.51078341 0.504862695 0.523842332 0.516200533 0.53400874

LASSO CN_high CN_low MSI PLOE CTNNB1 PIK3CA PIK3R1 PTEN

RF 0.904841195 0.904841195 0.891230094 0.859715822 0.879170168 0.820409474 0.810142933 0.79122248

ADABAG 0.791363698 0.791363698 0.795799373 0.698540707 0.781512605 0.705580088 0.702533333 0.697875871

DT 0.725246655 0.725246655 0.676739812 0.5 0.678518908 0.626926937 0.622 0.599717889

GBDT 0.863749155 0.863749155 0.86184326 0.808325653 0.814642857 0.695330189 0.708346667 0.710128333

LR 0.726860387 0.726860387 0.702751097 0.720514593 0.689956483 0.597372541 0.608653867 0.612167275

nNB 0.688917421 0.688917421 0.663009404 0.620583717 0.582247899 0.576535528 0.544266667 0.553330014

SVM 0.693674821 0.693674821 0.627774295 0.601382488 0.63125 0.564000401 0.583333333 0.57152893

KNN 0.529747263 0.529747263 0.529294044 0.521920123 0.513361044 0.525844039 0.515650667 0.50353413

RF CN_high CN_low MSI PLOE CTNNB1 PIK3CA PIK3R1 PTEN

RF 0.826081903 0.882343944 0.772797492 0.75422427 0.843643642 0.793456443 0.773341867 0.776362429

ADABAG 0.783146371 0.773501447 0.694545455 0.711597542 0.733321646 0.687143717 0.679733333 0.682459343

DT 0.701892148 0.641174039 0.618056426 0.5 0.652711547 0.610879165 0.604266667 0.578692333

GBDT 0.799151237 0.815502274 0.64565768 0.772273425 0.718016597 0.603362104 0.678961067 0.634039164

LR 0.790512231 0.70852005 0.6134721 0.645737327 0.607263908 0.555653352 0.638523733 0.538069477

nNB 0.689039059 0.673935511 0.557868339 0.563364055 0.538604488 0.510467684 0.548533333 0.530606262

SVM 0.707136099 0.640926002 0.511912226 0.5 0.5 0.54014452 0.5912 0.507384666

KNN 0.572501689 0.523317487 0.528263323 0.539938556 0.560956054 0.508665697 0.525245867 0.512650183

XGBoost CN_high CN_low MSI PLOE CTNNB1 PIK3CA PIK3R1 PTEN

RF 0.888595756 0.852563043 0.87017931 0.859577573 0.908066097 0.869682858 0.867665067 0.857625844

ADABAG 0.811001487 0.775775114 0.756677116 0.819815668 0.824672365 0.760437575 0.775466667 0.746000664

DT 0.716718475 0.641256718 0.677742947 0.532488479 0.676695157 0.654777198 0.6752 0.677088173

GBDT 0.822348966 0.790190161 0.803380564 0.849139785 0.844312251 0.789036532 0.7998336 0.791476933

LR 0.714491147 0.719210418 0.734129154 0.69609831 0.673385755 0.645403452 0.655424 0.667733156

nNB 0.679875659 0.666184374 0.660376176 0.632795699 0.555156695 0.607266158 0.5616 0.59909282

SVM 0.664238411 0.638838363 0.631473354 0.5 0.555042735 0.59937776 0.615466667 0.628974444

KNN 0.59986755 0.576800331 0.487789342 0.556973886 0.530539031 0.51514452 0.5474176 0.55285983
