# Supplementary material for: Integrative models of histopathological images and multi-omics data predict prognosis in endometrial carcinoma
Source: PeerJ. 2023 Aug 11;11:e15674. doi: 10.7717/peerj.15674 (PMC10424667; doi:10.7717/peerj.15674)
Supplement: Supplemental Information 6 — Cox regression analysis and Kaplan-Meier curves showed a significant difference in survival between high-risk and low-risk patients. [file peerj-11-15674-s006.docx]

Table S6: Predictive performance of prognostic modles.

| **Data category** | **HR** | **95%CI** | **P-value** |
| --- | --- | --- | --- |
| HIF | 9.32 | 6.08-13.16 | <0.001 |
| Genomics | 3.85 | 2.25-6.62 | <0.001 |
| Transcriptomics | 6.84 | 3.66-9.77 | <0.001 |
| Proteomics | 10.10 | 8.97-12.68 | <0.001 |
| Multi-omics model | 18.35 | 11.09-25.65 | <0.001 |

HIF, histopathologic image features; HR, hazard ratio; CI, confidence interval.
